# Miniaturized Digestion and Extraction of Surface Proteins from* Candida albicans* following Treatment with Histatin 5 for Mass Spectrometry Analysis

**DOI:** 10.1155/2016/9812829

**Published:** 2016-12-01

**Authors:** Shirley Fan, Eduardo B. Moffa, Yizhi Xiao, Walter L. Siqueira, Ken K.-C. Yeung

**Affiliations:** ^1^Department of Chemistry, Faculty of Science, University of Western Ontario, London, ON, Canada N6A 5B7; ^2^Department of Biochemistry, Schulich School of Medicine and Dentistry, University of Western Ontario, London, ON, Canada N6A 5C1; ^3^Schulich Dentistry, Schulich School of Medicine and Dentistry, University of Western Ontario, London, ON, Canada N6A 5C1

## Abstract

A common approach to isolate surface proteins from fungal and bacterial cells is to perform a proteolytic cleavage of proteins on the surface of intact cells suspended in solution. This paper describes miniaturization of this technique, in which cells are adhered on glass surfaces, and all sample treatments are conducted at *μ*L volumes. Specifically,* Candida albicans* cells were attached onto HSA-coated glass slides. By depositing the appropriate reagent solutions on the adhered cells, we successfully performed cell washing, treatment with antifugal peptide, Histatin 5, and a proteolysis on intact cells with trypsin. The resulting peptides were subsequently analysed by mass spectrometry. In general, the data obtained was similar to that collected with suspended cells in much larger sample volumes. However, our miniaturized workflow offers the benefit of greatly reducing the consumption of cells and reagents.

## 1. Introduction

*Candida albicans *is a very common fungus found within the genitourinary and gastrointestinal tracts, as well as on the surface of skins [1]. It exists within humans in a commensal relationship, where it benefits without causing damage [2]. However, individuals with compromised immune systems can develop the infection known as candidiasis [2, 3]. The most commonly used method to treat infections is to administer antifungal drugs orally or systematically into the circulatory system [2, 4]. Despite these mechanisms,* C. albicans *has developed resistance towards them [4], and thus, the discoveries of new therapeutic approaches are needed.

For infections to begin, adhesion to oral surfaces of protein films is the initial step [5, 6]. From these proteins, salivary proteins such as histatins, statherins, and acidic proline-rich ones have exhibited antifungal properties [7, 8]. The family known as histatins (HTN), comprised of HTN1, HTN3, and HTN5, have been of increasing interest due to its clear ability to control and kill* C. albicans *[5, 7, 9, 10]. Studies targeting the effects of HTN5 on cellular respiration at the mitochondrial level have been performed; however, the exact mechanism remains unknown [9, 11, 12]. Mass spectrometry- (MS-) based proteomics is therefore the next logical choice of method in studying* C. albicans* and its influence by HTN5.

MS is becoming an increasingly popular tool in studying microorganisms. Common applications include protein profiling for taxonomy classification, as well as clinical differentiation between pathogenic and nonpathogenic species [13–17]. The degree of classification achieved has been reported down to the genus, species, and strain levels [18]. Mass spectral protein profiling has also been incorporated into studying the response of bacterial cells to drug treatment [19]. Typically, the analysis of the total proteome, of lysed cells, is preferred for a comprehensive, nontargeted characterization. But the use of total cell lysates has its drawbacks. Due to the complexity of the proteome and background of lysed cells, sample cleanup and/or fractionation is crucial to obtaining high quality MS data [16]. The identification of a large number of proteins also requires a substantial effort in data analysis. Alternatively, the analysis of intact whole cells offers the benefit of reduced complexity. Anhalt and Fenselau were among the earliest to report the analysis of whole cells for species identification [20]. Through gentle heating in the ion source, phospholipids were vaporized, leading to the production of characteristic mass spectra. Alternatively, species identification through proteins and peptides from whole cell analyses has also been reported [21–23].

When applying MS to study changes in protein levels of* C. albicans* upon treatment with HTN5, the global approach of studying the total proteome was certainly appropriate. However, our group previously observed a reduction in cell adhesion property upon administration of HTN5 in 90 minutes, suggesting an effect of HTN5 on the surface proteins [24]. Hence, the targeted approach on the surface proteins is logical for studying the influence of HTN5. In 2006, Rodríguez-Ortega et al. reported a protocol called “cell shaving,” which allowed them to focus on proteins partially exposed on the outside of cells [25]. This was done by subjecting whole live cells to proteolytic enzymes in a solution. The surface peptides cleaved from intact cells were subsequently isolated for MS analyses. Others have also used a similar approach towards studying surface proteins from other fungal and bacterial species [26, 27]. Despite its effectiveness, the procedure of “cell shaving” is still generally time-consuming. Cells grown in media were harvested and washed, followed by overnight tryptic digestion for 18–20 hours in mL volume solution. The sample mixture was then centrifuged, and the supernatant was collected for fractionation and/or enrichment prior to injection into the liquid chromatography-mass spectrometer [28–30]. Furthermore, to overcome biological variation and growth variability, the entire procedure was typically repeated for multiple times, thus requiring large quantities of cells and reagents.

In this study, the aim is to refine and miniaturize the existing cell shaving technique, by performing the procedures on adhered* C. albicans* cells rather than suspended cells in solution. We will demonstrate that the attachment of cells on surfaces allows easy introduction and removal of *μ*L volume reagents solutions with a micropipette. Furthermore, treatment with antifungal peptide, HTN5, is readily performed on the adhered cells. We will also demonstrate that the *μ*L sample volume resulting from our miniaturized approach is directly compatible with subsequent MALDI MS and/or LC ESI MS/MS analyses. Importantly, the resulting MS data should be comparable to previous information obtained with the conventional, in solution, approach that required much larger quantities of reagent and cells.

## 2. Materials and Methods

### 2.1. *C. albicans* Culturing

The strain of* C. albicans *used for all experiments was ATCC90028. Stock cultures of cells were grown on plates containing Sabouraud dextrose agar (SDA) medium (Becton Dickson and Company, NJ, USA). The inoculated plates were incubated at 37°C for 48 hours. A third of a loop of the colonies were transferred into 45 mL of yeast nitrogen base (YNB), supplemented with 50 mM sucrose (Sigma Aldrich, MO, USA). The mixture was then incubated at 37°C for 21 hours under shaking at 200 rpm. Following the incubation, the mixture was centrifuged at 6000 rpm for 5 minutes. The pellet was resuspended in phosphate buffered saline solution (PBS, pH 7.4, Sigma Aldrich). The final cell suspension was stored at 4°C until use.

### 2.2. Adhesion of* C. albicans* to Glass Slides and Treatment with HTN5

**Figure 1:**
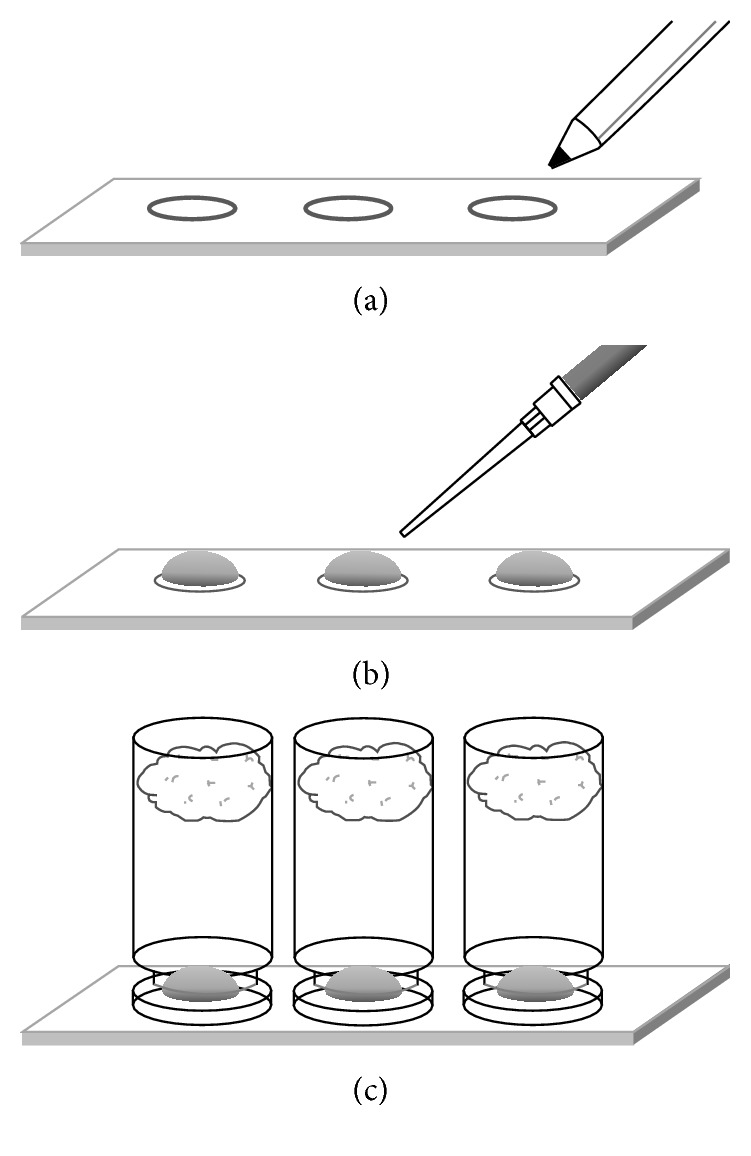
Schematics of the miniaturized procedures of cell adhesion, treatment, and tryptic digestion. Description is presented in Materials and Methods.

A Dako delimiting pen, composed of 60–100% 1-bromopropane and 5–10% dipentene (Cedarlane, Burlington, CA), was used to draw circles with an inner diameter of roughly 6 mm on standard microscope glass slides (Figure 1(a)). The hydrophobic ink functioned as a barrier to surround a droplet of aqueous sample solution. A total of three circles were made on each glass slide (Figure 1(b)). During an experiment, each sample spot was covered with an inverted glass vial containing a wet cotton, which maintained a high humidity environment and minimized evaporation of the sample droplet (Figure 1(c)). To prepare for cell adhesion, the glass surface within the hydrophobic circles was treated with 25 *μ*L of 0.10 mg/mL human serum albumin (HSA) solution (Sigma Aldrich) for 2 hours at room temperature (RT), based on a protocol previously reported by our group [5]. The HSA-coated surface was then washed three times with water. Next, 25 *μ*L of* C. albicans *suspension, at a concentration of 10^7^ cells/mL, was introduced to the HSA-coated slide. The adhesion was carried out under a 90-minute incubation at RT. The surface with adhered cells was then rinsed three times with PBS to remove the unattached cells. For the HTN5 treatment, 25 *μ*L of HTN5 solution (ChinaPeptides Company, Shanghai, China) at various concentrations was deposited to the cells. When not specified, a HTN5 concentration of 30 *μ*g/mL was used. The HTN5 treatment was done for 90 min at RT, which was the minimum time to observe a clear effect of HTN5 on* C. albicans* [24]. The final samples of treated cells on the glass slide were washed three times with water.

### 2.3. Tryptic Digestions on Solid Support

In a previous report, the trypsin digestion of surface proteins on* C. albicans* cells, suspended in buffer solution, was performed at a trypsin concentration of 10 *μ*g/mL trypsin for up to 20 min [28]. The authors also performed propidium iodide staining experiment to confirm that the* C. albicans* cells were not permeabilized by trypsin. In this work, digestions were performed on adhered* C. albicans* cells, with or without HTN5 treatment, on glass surfaces within the hydrophobic circles at RT. It was therefore necessary to reoptimize the digestion time and trypsin concentration. Due to the large number of experiments required to map these two parameters, we decided to first evaluate the digestion using a standard protein, cytochrome c from horse heart (Catalogue number C2506, Sigma Aldrich). For all digestions in this work, tosyl phenylalanyl chloromethyl ketone- (TPCK-) treated trypsin resuspended in 50 mM ammonium bicarbonate (Sigma Aldrich) was used.

To determine the optimal digestion time for cytochrome c, a very high concentration of trypsin was initially used to ensure that it was not a limiting factor. Specifically, 0.36 *μ*L of 0.1 mg/mL cytochrome c was mixed with 0.36 *μ*L of 1.0 mg/mL trypsin to perform the digestion directly on a MALDI sample target plate, under a range of digestion times from 0.25 to 60 min. After the optimal time was determined based on MALDI MS results, which turned out to be 10 min, the digestion was then performed on adhered* C. albicans* cells. The trypsin concentration used was reduced to a range comparable to that reported in the literature [28], namely, 0.5 to 2.0 *μ*g/mL, and a volume of 3 *μ*L was used. The optimal trypsin concentration was determined to be 1.5 *μ*g/mL based on MALDI MS results. Finally, using this trypsin concentration, we reexamined the digestion time study with adhered* C. albicans* cells. The MALDI MS results confirmed that the optimal digestion time remained at 10 min. Finally, following the digestion step performed on* C. albicans* cells, the trypsin solution, now with resulting peptides, was recovered using a micropipette for subsequent MALDI MS or LC ESI MS/MS analyses.

### 2.4. MALDI MS Analysis

The *α*-cyano-4-hydroxycinnamic acid (CHCA) matrix solution was prepared at 5.5 mg/mL in 6 mM ammonium phosphate monobasic, 50% acetonitrile, and 0.1% trifluoroacetic acid (Sigma Aldrich). The recovered peptides, 0.72 *μ*L from cytochrome c or 3 *μ*L from* C. albicans*, were mixed in a 1 : 1 (v/v) ratio with this CHCA matrix solution. All sample-matrix mixtures were spotted at a volume of 0.75 *μ*L on a 384 well Opti-TOF 123 × 83 mm SS MALDI plate (Sciex, MA, USA). The instrument is equipped with a 349 nm OptiBeam On-Axis laser with a pulse rate at 400 Hz. Data acquisition and processing were done using TOF-TOF Series Explorer (Sciex) and Data Explorer. The spectra were acquired in Reflectron positive mode from 500–3500* m/z*. Peak lists were created using the following parameters: a peak density of 10 per 25 Da, minimal signal-to-noise (S/N) of 10, minimum area of 50, and a maximum peak per spot of 200. Second fragmentation, MS/MS, was also performed via postsource decay (PSD) using the 1 kV in positive ion acquisition mode.

### 2.5. Nano-HPLC ESI MS/MS

Prior to injection for LC MS, the peptide sample (3 *μ*L) underwent sample cleanup using C_18_ ZipTip (Millipore, MA, USA). The eluted peptides were then dried and concentrated by the Vacufuge vacuum concentrator (Eppendorf, Germany). This was performed at RT for 10 minutes at 14000 rpm. Once dried, the peptides were resuspended in 10 *μ*L of 0.1% formic acid (Sigma Aldrich), with 8 *μ*L of this sample injected to LC MS.

Nano-HPLC was carried out on an Thermo Scientific Easy nLC II instrument (Thermo Scientific, CA, USA). The 85-minute gradient composition ranged from 5 to 55% of solvent B, which was 97.5% acetonitrile and 0.1% formic acid (Sigma Aldrich). Solvent A is 0.1% FA in water. The flow rate used was 200 nL/min at a pressure of 280 bar. The volume of sample injected was 8 *μ*L. ESI was conducted using a voltage of 2.0 kV with an ion transfer capillary temperature of 250.0°C.

MS analyses were performed on a Thermo Scientific linear trap quadrupole (LTQ) Velos ion mass spectrometer (Thermo Scientific). Positive mode data acquisitions and processing were done using Thermo Xcalibur 2.1.0 SP1.1162 software (Thermo Scientific, USA). The MS scan* m/z* range used was 0–2000. MS/MS was done via collision induced dissociation (CID) using helium (He) as the inert gas.

### 2.6. Database Searches

All MALDI MS spectra were searched against the Uniprot protein database (SwissProt 2015_02, 547 599 entries) on MASCOT provided by Matrix Science (http://www.matrixscience.com/). The search parameters used for* C. albicans *were (1) trypsin with one missed cleavage, (2) fungi as the taxonomy, (3) a variable modification for the oxidation of methionine, and (4) a mass tolerance of ±70 ppm.

All ESI MS/MS spectra were run through the Thermo Proteome Discoverer version 1.3.0.339 software (Thermo Fisher Scientific Inc., USA), using the algorithm known as SEQUEST. The* C. albicans *database chosen to search results against was the UniProt Knowledgebase, more commonly known as UniProtKB. (UniProt Consortium, http://www.uniprot.org/). The data analysis program SEQUEST was used for protein identification. The search parameters were as follows: (1) enzymatic cleavage by trypsin with up to 2 missed cleavages, (2) signal-to-noise ratio (S/N) of 1.5, (3) precursor mass tolerance of ±2 Da, and (4) fragment mass tolerance of ±0.8 Da.

## 3. Results and Discussion

### 3.1. *C. albicans* Cell Adhesion and HTN5 Treatment

Our research team previously studied and reported fungicidal activity of HTN5 on* C. albicans* [24]. The results indicated that preexposition of HTN5 to oral epithelial cells diminished the adhesion of* C. albicans* to the epithelium. Since the miniaturized assay in this work is performed on adhered cells, it is important to determine an optimal dosage of HTN5 which is sufficient to cause detected changes in protein abundance, but not too high to eradicate and desorb all cells from the glass surface.

**Figure 2:**
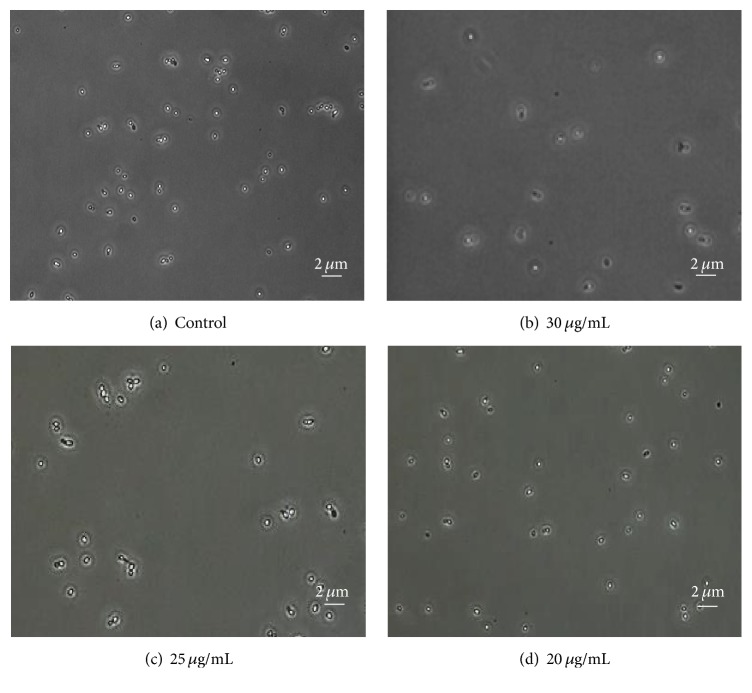
Microscope images of* C. albicans *adhered on slides after a 2-hour treatment with Histatin 5. The concentrations of HTN5 are indicated in (b–d) with units of *μ*g/mL. The average cell counts (*n* = 4) with standard deviations were (a) 110 ± 8, (b) 16 ± 8, (c) 25 ± 5, and (d) 42 ± 4.

To begin, we reproduced the adhesion of* C. albicans *on HSA-coated glass surfaces as previously reported [5], and then the cells were treated with varying concentrations of HTN5. Given the physiological concentration of HTN5 in the oral cavity, 10–30 *μ*M [31], we performed HTN5 treatments at 20, 25, and 30 *μ*g/mL (Figures 2(b) and 2(c)). A 2-hour treatment time was selected, based on the previous observation that 90 min was sufficient to cause a reduction in* C. albicans* colonization [24].  Figure 2 shows the typical microscopical views of the adhered cells after HTN5 treatments. The average cell count numbers presented in the caption refer to counts within the microscopical views. As expected, a reduction in cell counts after exposure to HTN5 was evident, and the effect was greater at higher HTN5 concentration. Given that sufficient cells remained adhered even at the highest concentration studied, 30 *μ*g/mL, we chose to use this concentration in subsequent HTN5 treatments to maximize changes in the* C. albicans* protein levels.

### 3.2. Tryptic Digestions Performed on Adhered Cells

Prior to performing digestion of* C. albicans *cells adhered on glass surfaces, an optimization of the tryptic digestion conditions was conducted. The first objective was to determine the shortest reaction time require to complete a digestion with an excess quantity of trypsin. We chose to conduct this study on a standard protein, cytochrome c. In contrast to working with cells, the use of a standard protein allows us to easily control the amount of starting materials and provides a set of well-defined tryptic peptide products as the reaction end-point. Triplicate digestions at RT were conducted under the concentrations of 0.05 mg/mL for cytochrome c and 0.5 mg/mL for trypsin, at a total volume of less than 1 *μ*L. The reaction time durations studied were 0.25, 0.5, 0.75, 1, 2, 3, 4, 5, 10, 15, 20, 25, 30, and 60 minutes. It was observed that the shorter digestion times of 15 seconds to 4 minutes produced considerably low peptide signals (data not shown). The most critical changes in number of peptides and/or peak intensities occurred between 5 and 15 minutes of digestion time. A total of eight tryptic peaks of cytochrome c, with up to 1 missed cleavage, were detected (*m*/*z* of: 634.39, 1168.62, 1260.58, 1296.72, 1454.81, 1495.70, 1598.78, and 1633.82). The changes in peak intensities of these eight peptides between digestion periods of 5 to 15 minutes are shown in Figure S1 (see Supporting Information in Supplementary Material available online at http://dx.doi.org/10.1155/2016/9812829). Essentially, highest signals were recorded from the digestion time of 10 minutes. Beyond 10 minutes, the digestion appeared to be completed, and the opposing effect of peptide loss due to nonspecific adsorption on surfaces was speculated.

Using the optimal digestion time of 10 minutes for cytochrome c, we subsequently optimized the trypsin concentration for the adhered* C. albicans *cells. The trypsin concentrations used for this experiment were 0.5, 0.75, 1, 1.5, and 2 *μ*g/mL. Five replicate digestion experiments were conducted by depositing 3 *μ*L of trypsin solution at various concentrations on adhered cells as shown in Figure 1. From the 25 resulting mass spectra, peaks with S/N above 10 were extracted for their values of *m*/*z* and peak height. Background signals from trypsin and HSA were removed from the list, and averages of peak intensities were taken from replicates. The two lowest concentrations studied, 0.5 and 0.75 *μ*g/mL, resulted in noticeably fewer peaks, and so only the results from the higher three concentrations were shown in Table  S1 (under Supporting Information). Based on the number of signals observed, our results indicated that 1.5 *μ*g/mL was the optimal. In addition, the peak intensities were also highest at this concentration in most cases.

Once again, the optimal digestion time of 10 min was previously determined using cytochrome c. In case this optimal value is substrate-specific, we reexamined the effect of digestion time for* C. albicans* at a trypsin concentration of 1.5 *μ*g/mL. Four digestion times, 5, 10, 15, and 20 minutes, in replicates of four were studied. The results are presented in Table  S2, which confirmed that 10 minutes remained to be optimal in yielding the highest number of signals. Previous work performed the isolation of surface peptides from* C. albicans* in solution under digestion times from five to twenty minutes [28]. They reported that five minutes was sufficient for the release of easily accessible surface peptides, and as the time was increased, the number of resulting peptides increased as well. Our data generally agreed with their observations. Even though the greatest number of peaks was observed at 10 minutes, the peak intensities of some peaks continued to increase beyond 10 minutes, for example, *m*/*z* of 656.0, 851.4, 1111.5, and 2447.0. However, we have not yet confirmed which of these signals originated from* C. albicans*, and thus the comparison should not be based on the intensities of selected peaks. This led us back to the conclusion of 10 minutes being optimal based on the highest number of observed peaks.

Most importantly, the results demonstrated the successful tryptic digestion performed on* C. albicans *adhered on glass, with as little as 3 *μ*L of trypsin solution. Using the established optimal reaction time of 10 min and trypsin concentration of 1.5 *μ*g/mL, digestions were performed finally on* C. albicans *cells treated with HTN5. The resulting peptides were recovered for analysis by LC ESI MS/MS for identification.

### 3.3. Analyses of Peptides from* C. albicans* by Nano-HPLC ESI MS/MS

ESI MS and MS/MS data were generated for samples collected from the tryptic digestion of* C. albicans* cells, with and without HTN5 treatments. The entire study was performed in replicates of six for each of the two groups, and the results were searched against the* C. albicans *database for protein identification. It is noteworthy that the nature of our miniaturized assay, while reducing the consumption of reagents and cells, also produces a limited quantity of peptides for protein identification. It was estimated that less than 2 *μ*g of materials was injected for each LC MS analysis. For this reason, we have taken a less stringent approach in setting our database search parameters, and so we can include results with moderate to high levels of confidence. Furthermore, instead of focusing only on peptides with substantial changes in abundance after exposure to HTN5, we also take the inclusive approach and present the different levels of abundance changes resulting from the treatment. To help readers distinguishing the peptides detected with increased intensities from those with reduced intensities following HTN5 treatment, the results are divided into two tables.  Table 1 presents the precursor ions with average intensities that were higher from the HTN5 treated samples compared to the control, whereas Table 2 shows the ions with lower intensities from the treated group compared to the control. The *p* values resulting from *t*-test are included in the tables to illustrate the significance of the signal intensity changes. Entries with high *p* values (>0.1) should be treated as statistically indistinguishable. The signal intensities of these ions were highly variable within the replicate measurements, which resulted in large standard deviations and low statistical confidences.

The confidences of protein assignments were also illustrated in the tables as *X*_Corr_ values. *X*_Corr_ values above 2 are usually indicative of a good correlation. It is noteworthy that some observed precursor ions were matched with multiple theoretical precursor ions; for example, observed *m*/*z* 1297.59 was matched with theoretical *m*/*z* 1298.54, 1297.49, 1297.69, 1297.70, 1297.75, and 1298.67. Readers should take into account the *X*_Corr_ scores when interpreting the results, as some of these protein assignments are only putative. Finally, the protein entries were sorted according to their biological functions, but these listed functions were taken straight out of the Uniprot database based on the accession numbers resulting from the search (listed next to the protein names in Tables 1 and 2). Further experimental verifications were not conducted in this work.

In the previous study of peptides resulting from cell shaving of* C. albicans *in solution [28], the authors reported the detection of proteins with the following functions: metabolism, cell defense and virulence, transport, and protein fate. Likewise, the profiling of* C. albicans *surface proteins was reported by two other groups in different analytical approaches [29, 32]. Broadly speaking, similar functions were observed in Tables 1 and 2. While these previous reports offered more comprehensive listings of proteins with cell wall functions, our work focused on the changes in surface proteins upon HTN5 treatment.

In another previous study, quantitative LC MS was performed to characterize changes in the mitochondrial proteome of* C. albicans* upon HTN5 treatments [33]. The authors reported an upregulation of mitochondrial proteins mainly involved in genome maintenance and gene expression and a downregulation for respiratory enzyme complexes. In our work, the MS analysis was performed on samples resulted from the tryptic shaving of adhered intact cells. Given that the two approaches focused on different parts of the proteome, we should not directly compare the proteins identified from these studies. It suffices to conclude that a general decrease in MS signals was observed in our work for many virulence- and surface-associated proteins after HTN5 treatment, as one would expect. Finally, readers should be reminded that the peptide sample quantity produced from our miniaturized technique was substantially lower than that typically used in previous studies, due to the lower amount of starting materials in our work. Nevertheless, one could alleviate this limitation by increasing the density of adhered cells on glass with a cell suspension of higher concentration and/or by increasing the surface area used of each assay, which is defined by the hydrophobic boundary made by the user. An increase in cell number per assay will directly increase the quantity of peptides produced for LC MS analysis and subsequently improve the number of proteins identified and/or the confidence of the identification.

## 4. Conclusions

This paper reported successful miniaturization of the tryptic digestion of surface proteins performed on whole cells. In particular,* C. albicans* were adhered on glass surface, where introduction and removal of reagent solutions were conveniently conducted with a micropipette. Compared to the use of cells suspended in solutions, our treatment of adhered cells also better resembles the antifungal treatment of* C. albicans* cells adhered on tissues in the oral cavity. Importantly, the reduction in reagent volumes from mL to *μ*L levels will allow users to perform many more replicate assays, while keeping an overall low consumption of reagents and cells. Nevertheless, the downside of the miniaturization is the reduced peptide sample quantity resulting from the LC MS analysis, which could reduce the confidence of the protein identification. Ultimately researchers should select methods based on their purposes. In this case, the miniaturized approach with adhered cells is more effective for high-throughput screenings of cell types or fungicides, while the batch mode with suspended cells is more suitable for a comprehensive profiling of the proteome.

## Supplementary Material

A series of experiments were performed to determine the optimal conditions for the tryptic digestion of *C. ablicans* cells adhered on glass. To begin, the digestion time was optimized for a standard protein, Cytochrome c, with an excess amount of trypsin (Figure S1). Next, under this optimal digestion time, the trypsin concentration was optimized for *C. ablicans* cells adhered on glass (Table S1). Finally, we confirmed the optimal digestion time by repeating the experiments using the optimized trypsin concentration for *C. ablicans* cells (Table S2).

## Figures and Tables

**Table 1:** Proteins identified from a miniaturized assay of HTN5 treatment of *C. albicans* with decreased MS signals. Average MS signals and their standard deviations were calculated based on six replicates. Proteins indicated with an asterisk (*∗*) have been determined using their associated protein families. Lower case letters in amino acid sequences indicated modified residues. Blank spaces indicated proteins unidentified by the databases.

Biological function	Observed precursor *m/z*	Theoretical precursor *m/z*	MS signal intensities		*p* values	Amino acid sequence	Protein name (UniProt Accession Number)	Charges	*X*_Corr_
			Control	HTN5 treated					
Virulence	1284.83	1286.65	27000 ± 9500	14000 ± 2800	0.016	K.sLYTISPNKGK.R	Transcriptional regulator STP3 (A0A0A6LWB8)	3	2.52
		1283.69				K.SKYNLPFAMK.E	Candidapepsin-7 (A0A0A4B714)		3.75
	1295.87	1295.70	66000 ± 1100	19000 ± 7000	0.000013	R.tKKWGLGWIK.N	Integrase family^*∗*^ (A0A0A3BML7)	1	1.61
	3579.79	3581.47	40000 ± 5000	31000 ± 36000	0.30	HPQYsEACsAVmVVTYSSGSGEHIH TTDIK	Kexin (A0A0A6IYX0)	3	3.33
	3895.36	3892.80	36000 ± 4100	26000 ± 3100	0.0037	ELKTTVIVTSCFNNVCSETsITT PKtAVtATtSK	Flocculin^*∗*^ (A0A0A3CM71)	3	3.10
	4659.96	4658.22	15000 ± 1100	3200 ± 2700	0.00014	K.HLTLKSSTPASTLEYSTSIPPALATT SSSLStESTtLttISR.S			3.25
	4115.51	4115.94	51000 ± 12000	24000 ± 17000	0.017	VTFVEKAtSTSTTNtTttTTTTTTTTTT TTTIPVKR	*β*-Lactamase family^*∗*^ (A0A0A6K6B3)	3	3.23
	4121.03	4122.05	31000 ± 23000	17000 ± 7200	0.13	K.NVKVITTTTTtSPSSFSSSSSLMsPITPQT PNIPKTPK.T	PX domain^*∗*^ (A0A0A3CBE8)	3	3.94

Surface-associated	1175.88	1173.66	64000 ± 3900	22000 ± 1100	<0.00001	R.tINLDsQVK.Y	3′(2′),5′-Bisphosphate nucleotidase 2 (A0A0A6NTV1)	1	3.16
	4659.96	4659.87	66000 ± 1100	20000 ± 7000	0.00001	mQTSISttTIEDHLHHYsPEESQKLLSRE SSINTDLFK.E	Bud site selection protein BUD4 (A0A0A3DIG6)	3	3.13

Mitochondrial-associated	1175.88	1175.15	64000 ± 3900	22000 ± 1100	<0.00001	K.YMLLTLLtK.L	YAP-bd/ALF4/glomulin family^*∗*^ (A0A0A3CAC1)	1	2.96
	1295.87	1297.68	66000 ± 1100	19000 ± 7000	0.00001	K.LTtLISSIENK.I			2.96
	1374.01	1376.70	16000 ± 1300	10000 ± 3000	0.0047	R.TASGNIIPSSTGAAK.A	Glyceraldehyde-3-phosphate dehydrogenase (Q92211)	1	2.86
	4659.96	4660.23	15000 ± 1100	3200 ± 2700	0.00014	K.StIVEEIYsNARSHLVQGNKEmGmALFNE LLAINESIYGK.V	Clustered mitochondria protein homolog (A0A0A4C6G1)	3	3.37

DNA binding	804.77	804.44	42000 ± 22000	19000 ± 3100	0.034	K.QPAVFSR.I	DNA polymerase (Q5A4E5)	1	2.56
	1284.83	1283.53	27000 ± 9500	14000 ± 2800	0.016	R.VASHsLsTsR.R	Minichromosome maintenance protein 10 (A0A0A3CIN8)	1	2.67
	3331.28	3331.42	63000 ± 1200	14000 ± 1400	<0.00001	K.GFTNTMISHIGFDPTGtsLNQNsTS LGsK.S		3	3.21
	2806.76	2806.20	47000 ± 36000	30000 ± 23000	0.21	R.TTPPTVsITGPNPsSSPAsASTNtSK.S	Phosphatase family^*∗*^ (A0A0A3BQA3)	3	3.60
	3376.60	3376.98	43000 ± 12000	32000 ± 28000	0.23	K.SSPTSsATTTATtSVsISSLSLtMGKPKNSK.L	Mediator complex^*∗*^ (A0A0A3CP70)	3	3.15
	3498.15	3500.80	34000 ± 8900	18000 ± 16000	0.054	K.LGSVLTtRSQLIEYELtTRtIFINCSA ALK.I	Spt6 (D3IZV2)	3	3.11
	3503.40	3504.74	43000 ± 8300	31000 ± 2500	0.014	R.EVIIKLSITRtHtPPPDSTtTTTPTTSIEK.T	Zn finger domain^*∗*^ (A0A0A6J6A5)	3	3.57
	3939.14	3940.46	36000 ± 4000	17000 ± 8000	0.0026	VSSYILDGNNSTKLPsPVLtHtTF DSRsDEGQR	Basic leucine zipper domain^*∗*^ (A0A0A3DLJ5)	3	3.99
	4115.51	4116.95	51000 ± 12000	24000 ± 17000	0.017	R.APQsIQLPPIQsFtKsQAVFPQSVRDSAPA ANFNR.Y	Transcription factor and DNA binding protein families^*∗*^ (A0A0A6ITE8)	3	3.12
	4659.96	4659.15	15000 ± 1100	3200 ± 2700	0.00014	MKImmIPTHHQtYNINTHQPPQQHQYLPPPGt SYTSPR.A			3.12
	4115.51	4114.87	51000 ± 12000	24000 ± 17000	0.017	K.sStQMSSCtNsVTQTLDRLPKIVSTQQNNL TPTSK.I	Zn(2)-DNA binding domain^*∗*^ (A0A0A6KY81)	3	3.03
	4659.96	4660.19	15000 ± 1100	3200 ± 2700	0.00014	R.RSVSYSPGPsSIKSQLPHLTSSSTTtssVQSPP PPPPSQPPR.G			3.61
	4173.60	4173.73	24000 ± 1200	13000 ± 1200	0.000014	K.tVTsINGSPPPLEtAPsSHHNVPIDFIHFK KESDR.T	Uncharacterized protein (A0A0A3EPD4)	3	3.12
	4368.59	4367.89	33000 ± 21000	7100 ± 1300	0.020	K.NMQFPPYQVSsHNsSEtSQsIPNTPSITRQVE SNTR.S	Transcriptional regulatory protein LEU3 (A0A0A3BTY8)	3	3.54
		4367.90				K.tPTTTTTTTTtANGNTSNGNTSNGNsTGKTATA ATATKSNtK	Transcription protein family^*∗*^ (A0A0A3C3Y1)		3.19

Protein synthesis	804.77	805.41	42000 ± 22000	19000 ± 3100	0.034	K.QTSLNDK.C	Mannosyltransferase family^*∗*^ (A0A0A3CUB4)	1	2.54
		804.45				R.VKIDSDK.S	Protein transporter SEC24 (A0A0A3EHG4)		1.51
	1175.88	1173.98	64000 ± 3900	22000 ± 1100	<0.00001	K.TKQFNDsKK.K	Vesicle tethering protein family^*∗*^ (A0A0A6KIG6)	1	2.01
	1284.83	1283.72	27000 ± 9500	14000 ± 2800	0.016	K.tAGsNHNsEK.K	Ribosomal RNA-processing protein 12 (A0A0A4BGJ7)	1	2.51
	4121.03	4119.93	31000 ± 23000	17000 ± 7200	0.13	K.FFsDNIANDLAtTTTTTTTTNTGAtSVHPIL QVDAIK.Y	CAS/CSE protein family^*∗*^ (A0A0A6L8P2)	3	3.24
	4368.59	4370.93	33000 ± 21000	7100 ± 1300	0.020	K.ESSSTADQPSVVPPQESHKDTVETPK PEVtEtsVEAtK.E	Translation initiation factor 4G (A0A0A4CDK4)	3	2.99
		4367.03				K.NPTPTPTPTPTPTPNNLAQGVDsSSTLDV EtTLtGLtRR.I	Imidazoleglycerol phosphate synthase cyclase subunit (A0A0A6I238)		3.21

Signalling	3503.40	3504.60	43000 ± 8300	31000 ± 2500	0.013	K.RSSITtPtPPLTTTHSSNGNGNGNV NVNVNsK.R	Peroxisome transmembrane receptor^*∗*^ (A0A0A3ZWC2)	3	3.30
	4115.51	4116.76	31000 ± 20000	17000 ± 7200	0.017	K.QSTTNTsTLssTtAASTLATSNNTQPD TYTSTSTSIR.G	Pleckstrin homology domain^*∗*^ (A0A0A6L5L9)	3	3.33
	4416.51	4414.87	29000 ± 27000	16000 ± 1100	0.10	R.QHPDPLSNQsNFNsNTINNYSNYRS sTRSGLDPsQR.H	Rho GTPase activating protein domain^*∗*^ (A0A0A3BMU5)	3	4.12
	4659.96	4660.32	15000 ± 1100	3200 ± 2700	0.00014	K.DLPIGYILHmINLcPNIVsLNLGNLSLsTD YEISRSTIHK.Y	F-box protein COS111 (A0A0A3CYY6)	3	3.68
		4658.21				K.WNKEKIELDsPLIVSYVSSLCNGGGGGIIT NsTNSTttNSK	Pentatricopeptide repeat (PPR) protein family^*∗*^ (A0A0A3CGE7)		1.11

Miscellaneous	1284.83	1285.42	27000 ± 9500	14000 ± 2800	0.016	R.NDsDtsLsK.E	Insulin induced protein family^*∗*^ (A0A0A3CRM0)	1	1.66
						R.YtmNEVFK.V	GYF domain^*∗*^ (A0A0A3C0N8)		
	3331.28	3331.59	63000 ± 1200	14000 ± 1400	<0.00001	ETsQFMGNAESEDLtGNGLLTSTL AVLSSIS	Orf in *C. albicans* major repeat sequence, RB2 region (Q5A475)	3	3.15
	3331.28	3332.44	63000 ± 1200	14000 ± 1400	0.23	K.LSSLGNHGTtTTSSLSSSsSsSIsNNT SIAK.I	CBS domain^*∗*^ (A0A0A6JTB4)	3	3.46
	3376.60	3376.59	43000 ± 12000	33000 ± 28000		R.KQQDQNEVAGAAAATTTTAtAtAT AATNWKPK.N			3.21
	3498.15	3498.57	34000 ± 8900	18000 ± 16000	0.054	K.SSsLIKNsTsSNQSsPATSTNTSIVDVPIEK.S	WD repeat protein^*∗*^ (A0A0A3CA48)	3	3.21
	4173.60	4172.95	24000 ± 1200	13000 ± 1200	0.000014	R.INNNNDKssILsNITTTNTTTGTGTTNTtT VPSIKTK.R	(A0A0A6IY53)		4.24
	3498.15	3499.48	34000 ± 8900	18000 ± 16000	0.054	KAFSSsKLTSDSANStNStNsTSMSILGNDK.D	CDK inhibitor PHO81 (A0A0A6MY31)	3	3.16
	3895.36	3894.86	36000 ± 4100	26000 ± 3100	0.0037	R.ISRPNGVGGISTSGSSSPTTEFVTPQAs KsSVDQNKK.R	Ubiquitin interacting motif^*∗*^ (C4YNW3)	3	3.29
	4421.03	4421.09	32000 ± 19000	17000 ± 7200	0.080	R.HLILGYKItVVtDHQSLTsVMTSSSRPE NNRMIR.W	Aspartic peptidase family^*∗*^ (A0A0A4CVF8)	3	3.83
	3939.14	3940.16	36000 ± 24000	17000 ± 18000	0.0026	R.NVNGSGStNtNTMtRLDsTTIASSLFCRQLYF NLLSK.D	Globin family^*∗*^ (A0A0A4CD75)	3	3.11
	4372.57	4373.90	9600 ± 4600	5400 ± 1800	0.058	R.NNsTVSSTNSLmSNNsDTNtAATAATAATSGS TTNNVKR.M	(A0A0A3XE46)		3.38
	4416.15	4414.82	29000 ± 27000	16000 ± 1100	0.10	R.NGtYSSsStSSSsTSSVSSSSATTANGESLNST THNIQLER.T	Sec2p^*∗*^ (A0A0A6JGN5)	3	2.89
	4659.96	4661.33	15000 ± 1100	3200 ± 2700	0.00014	K.QNYVDPGQIAIKGLKGFVNLGAtCFMssILQtLIHN PLI.K.Y	Ubiquitin carboxyl-terminal hydrolase (C4YKS7)	3	3.69
		4660.93				K.YDKPmEDTEEIDDVtSISKsINEQID DPFsQFNSVTLR.Y	Protein-serine/threonine kinase (A0A0A4ANB2)		3.51

Uncharacterized	1175.88	1173.73	64000 ± 4000	22000 ± 1100	<0.00001	K.tImtItIK.I	(Q59T39)	1	2.07
	3331.28	3332.60	63000 ± 1200	14000 ± 1400	<0.00001	R.IsNIITLNSSLSSSsSsSSSsSSLLLL TLK.S	(Q5AAP9)	3	3.11
	3895.36	3893.70	36000 ± 4100	26000 ± 3100	0.0034	K.TTPVMGNSStPSTVtANtNtGAYSTSSDTA AKPTKK.A	(A0A0A6JKT0)	3	3.14
	3939.14	3940.74	36000 ± 24000	17000 ± 18000	0.0026	K.TTPVMGNSStPSTVtANtNtGAYSTSSDTA AKPTKK.A	(A0A0A6JKT0)	3	3.46
	4115.51	4116.79	31000 ± 20000	17000 ± 7200	0.017	K.TVCNWQtLGHTDEFEEstQFARGVSDtAL VGGITK.L	(Q59K01)	3	2.18
		4114.82				T.CGKSCFINSttANKLIsYNLFQSStEVDS IGPTGSK.T	(Q59KZ4)		3.86
		4116.2				K.TTPVMGNSsTPSTVTANTNTGAYSTSsDt AAKPTKKATK.R	(A0A0A6JKT0)		3.91
		4115.04				R.ILLLLPLLGVtILTSKSSLESISLNsPSDSI ALssSK.S	(Q5AH83)		2.34
	4416.51	4415.18	29000 ± 27000	16000 ± 1100	0.10	K.STSSANIKtKPKPNTATtATTPTAtTATTtA TTSSIDPTEK.D	(A0A0A4CH02)	3	2.59
	4421.03	4419.99	32000 ± 19000	17000 ± 7200	0.080	R.LSGtNNPGsGsGsGGGGGGGANNNSLPGY SVGSSIGRGRGLGR.G	(A0A0A6LWB8)	3	1.89
	4368.59	4366.93	33000 ± 21000	7100 ± 1300	0.020	K.NYNQFKLIELDDSMNTTTTTTTTTTtTttT tTSTIGK.F	(A0A0A6MD82)	3	3.76
		4367.87				K.SKsTTIHNQSNIHSEQISINDENNNKStstS TSTDTK.K	(A0A0A3EQM3)		1.32
	4372.57	4370.82	9600 ± 4600	5400 ±1800	0.058	K.SSGGSSDTKSVWIAtTGSDFASQSNSDSs StASRNSSSSASR.Q	(A0A0A3BP13)	3	2.46
		4373.17				K.EsEGVWGWsLQCLLLLFTKVNLRscDQL LICALVIR.E	(Q5AKS1)		2.99
	4659.96	4661.36	15000 ± 1100	3200 ± 2700	0.00014	K.VILIGNSMTsRTTsSVRFFSIMLTVSLPItN ILVsSELSK.V	(A0A0A3BZ01)	3	3.11

**Table 2:** Proteins identified from a miniaturized assay of HTN5 treatment of *C. albicans* with increased MS signals. Average MS signals and their standard deviations were calculated based on six replicates. Proteins indicated with an asterisk (*∗*) have been determined using their associated protein families. Lower case letters in amino acid sequences indicated modified residues. Blank spaces indicated proteins unidentified by the databases.

Biological function	Observed precursor *m*/*z*	Theoretical precursor *m*/*z*	Ion counts		*p* values	Amino acid sequence	Protein name	Charges	*X*_Corr_
			Before	After					
Surface-associated	1297.59	1298.54	18000 ± 8800	51000 ± 5400	0.00042	K.KPsTEDtFSK.Y	Peroxisomal protein family^*∗*^ (A0A0A6JC81)	1	1.82

Mitochondrial-associated	1297.59	1297.49	18000 ± 8800	51000 ± 5400	0.00042	R.NsIttEsVK.A	Aldehyde hydrogenase family^*∗*^ (A0A0A3BNV6)	1	2.03

DNA binding	575.44	576.45	9700 ± 1000	28000 ± 6200	0.00055	K.SLLSR.I	Telomerase reverse transcriptase family^*∗*^ (C4YDL3)	1	1.54
	1297.59	1297.75	18000 ± 8800	51000 ± 5400	0.00042	K.NVIPAtItK.V	Spt6 (D3IZV2)	1	1.54
		1298.67				K.EFTIGPFKcIK.W	Transcriptional regulatory protein family^*∗*^ (A0A0A3CXJ5)		1.56

Miscellaneous	575.44	576.31	9700 ± 1100	28000 ± 6200	0.00055	R.SLNSR.I	ATPase family^*∗*^ (A0A0A4CD62)	1	3.15
	1901.71	1903.74	7800 ± 3900	13000 ± 2300	0.025	K.NKATssSSStsRDTR.W	Leucine rich repeats^*∗*^ (C4YMX3)	2	3.19

Uncharacterized	1297.59	1297.69	18000 ± 8800	51000 ± 5400	0.00042	R.NNTtVSKGRIK.V	(A0A0A6M207)	1	1.51
		1297.70				K.KHQtHILNVK.S	(A0A0A6HZ28)		1.60
