# Supplementary material for: Miniaturized Digestion and Extraction of Surface Proteins from Candida albicans following Treatment with Histatin 5 for Mass Spectrometry Analysis
Source: Int J Proteomics. 2016 Dec 1;2016:9812829. doi: 10.1155/2016/9812829 (PMC5156812; doi:10.1155/2016/9812829)
Supplement: Supplementary file 1 — A series of experiments were performed to determine the optimal conditions for the tryptic digestion of C. ablicans cells adhered on glass. To begin, the digestion time was optimized for a standard protein, Cytochrome c, with an excess amount of trypsin (Figure S1). Next, under this optimal digestion time, the trypsin concentration was optimized for C. ablicans cells adhered on glass (Table S1). Finally, we confirmed the optimal digestion time by repeating the experiments using the optimized trypsin concentration for C. ablicans cells (Table S2). [file 9812829.f1.docx]

Supporting Information

**Miniaturized Digestion and Extraction of Surface Proteins from *Candida albicans* following Treatment with Histatin 5 for Mass Spectrometry Analysis**

**Shirley Fan^1^, Eduardo B. Moffa^2,3^, Yizhi Xiao^2,3^, Walter L. Siqueira^2,3^ and Ken K. -C. Yeung^1,2^**

*^1^Department of Chemistry, Faculty of Science, University of Western Ontario, London, ON, Canada N6A 5B7*

*^2^Department of Biochemistry, Schulich School of Medicine and Dentistry, University of Western Ontario, London, ON, Canada N6A 5C1*

*^3^Schulich Dentistry, Schulich School of Medicine and Dentistry, University of Western Ontario, London, ON, Canada N6A 5C1*

A series of experiments were performed to determine the optimal conditions for the tryptic digestion of *C. ablicans* cells adhered on glass. To begin, the digestion time was optimized for a standard protein, Cytochrome c, with an excess amount of trypsin (Figure S1). Next, under this optimal digestion time, the trypsin concentration was optimized for *C. ablicans* cells adhered on glass (Table S1). Finally, we confirmed the optimal digestion time by repeating the experiments using the optimized trypsin concentration for *C. ablicans* cells (Table S2).

**Figure S1**. The MALDI MS peak intensity of eight tryptic peptides from the on-target digestion of cytochrome c under three digestion time periods. The intensity values shown were averages of triplicates.

**Table S1.** The mass and intensity of peaks detected by MALDI MS from the on-target digestion of *C. albicans* under three trypsin concentrations. The intensity values shown were averages of five replicates.

| **0.0010 mg/mL** | |  | **0.0015 mg/mL** | |  | **0.0020 mg/mL** | |
| --- | --- | --- | --- | --- | --- | --- | --- |
|  |  |  |  |  |  |  |  |
| m/z | Intensity |  | m/z | Intensity |  | m/z | Intensity |
|  |  |  |  |  |  |  |  |
| 529.2 | 2703 |  | 501.2 | 3589 |  | 506.1 | 15909 |
| 565.0 | 2216 |  | 511.8 | 3153 |  | 512.1 | 5356 |
| 580.9 | 1908 |  | 517.8 | 2647 |  | 524.1 | 15012 |
| 586.9 | 2958 |  | 564.9 | 18498 |  | 550.0 | 12520 |
| 602.9 | 2768 |  | 580.9 | 13835 |  | 568.0 | 32242 |
| 656.0 | 11816 |  | 584.2 | 2568 |  | 581.1 | 3804 |
| 659.3 | 68813 |  | 586.9 | 14077 |  | 599.4 | 24812 |
| 672.0 | 7377 |  | 596.9 | 6588 |  | 614.3 | 4189 |
| 721.3 | 2895 |  | 602.9 | 11396 |  | 617.0 | 7732 |
| 805.4 | 6396 |  | 618.9 | 3322 |  | 637.2 | 12715 |
| 1758.6 | 4086 |  | 655.2 | 3019 |  | 650.2 | 7235 |
| 1862.9 | 2936 |  | 656.0 | 69321 |  | 656.0 | 14746 |
| 1900.8 | 2385 |  | 659.3 | 65833 |  | 659.3 | 29891 |
| 2162.9 | 7411 |  | 672.0 | 40046 |  | 667.2 | 8156 |
| 2184.9 | 3457 |  | 674.0 | 2331 |  | 805.3 | 4142 |
| 2192.9 | 12182 |  | 677.2 | 4114 |  | 1020.4 | 5059 |
| 2273.0 | 20655 |  | 681.3 | 6966 |  | 1175.4 | 3009 |
| 2295.0 | 3369 |  | 688.0 | 8268 |  | 1433.5 | 2524 |
| 2304.9 | 9011 |  | 699.3 | 6022 |  | 1758.6 | 3930 |
| 2322.9 | 3596 |  | 703.3 | 6350 |  | 2162.9 | 14883 |
| 2326.9 | 1698 |  | 721.3 | 7632 |  | 2184.7 | 6089 |
| 2334.9 | 4213 |  | 728.9 | 3205 |  | 2192.8 | 13010 |
| 2336.9 | 1003 |  | 737.2 | 2137 |  | 2272.9 | 15580 |
| 2366.8 | 3553 |  | 744.8 | 2701 |  | 2304.8 | 11451 |
|  |  |  | 797.9 | 6051 |  |  |  |
|  |  |  | 805.4 | 4444 |  |  |  |
|  |  |  | 813.9 | 5769 |  |  |  |
|  |  |  | 867.0 | 3002 |  |  |  |
|  |  |  | 928.4 | 3533 |  |  |  |
|  |  |  | 950.4 | 3026 |  |  |  |
|  |  |  | 1059.4 | 3854 |  |  |  |
|  |  |  | 1081.4 | 4666 |  |  |  |
|  |  |  | 1161.4 | 2355 |  |  |  |
|  |  |  | 1175.5 | 3174 |  |  |  |
|  |  |  | 1758.6 | 7247 |  |  |  |
|  |  |  | 1862.9 | 3278 |  |  |  |
|  |  |  | 1900.7 | 2800 |  |  |  |
|  |  |  | 2162.9 | 6404 |  |  |  |
|  |  |  | 2184.9 | 3684 |  |  |  |
|  |  |  | 2192.8 | 13127 |  |  |  |
|  |  |  | 2214.8 | 2721 |  |  |  |
|  |  |  | 2273.0 | 19575 |  |  |  |
|  |  |  | 2295.0 | 4610 |  |  |  |
|  |  |  | 2304.9 | 11218 |  |  |  |
|  |  |  | 2322.9 | 4779 |  |  |  |
|  |  |  | 2326.9 | 2971 |  |  |  |
|  |  |  | 2366.8 | 2203 |  |  |  |

**Table S2.** The mass and intensity of peaks detected by MALDI MS from the on-target digestion of *C. albicans* under four digestion time periods. The intensity values shown were averages of five replicates.

| **5 min** | |  | **10 min** | |  | **15 min** | |  | **20 min** | |
| --- | --- | --- | --- | --- | --- | --- | --- | --- | --- | --- |
|  |  |  |  |  |  |  |  |  |  |  |
| m/z | Intensity |  | m/z | Intensity |  | m/z | Intensity |  | m/z | Intensity |
|  |  |  |  |  |  |  |  |  |  |  |
| 503.3 | 8355 |  | 517.2 | 5278 |  | 524.1 | 5152 |  | 506.1 | 5818 |
| 506.1 | 16024 |  | 537.3 | 5248 |  | 550.1 | 7398 |  | 524.1 | 8910 |
| 512.4 | 19758 |  | 550.6 | 6717 |  | 550.6 | 5148 |  | 530.2 | 6768 |
| 519.3 | 9617 |  | 554.3 | 7462 |  | 554.2 | 11942 |  | 550.1 | 9454 |
| 522.1 | 7406 |  | 564.9 | 4024 |  | 568.1 | 15210 |  | 554.2 | 6834 |
| 522.6 | 8989 |  | 574.9 | 3096 |  | 573.3 | 4142 |  | 568.1 | 23587 |
| 524.1 | 29471 |  | 580.9 | 7730 |  | 599.5 | 5079 |  | 573.2 | 4123 |
| 526.1 | 5249 |  | 596.9 | 8022 |  | 637.2 | 9484 |  | 599.5 | 5119 |
| 550.1 | 25141 |  | 602.9 | 4284 |  | 656.0 | 47208 |  | 617.0 | 5066 |
| 550.6 | 58727 |  | 612.9 | 5082 |  | 659.2 | 28094 |  | 637.2 | 9442 |
| 568.1 | 73810 |  | 618.9 | 5299 |  | 672.0 | 27411 |  | 656.0 | 29725 |
| 599.5 | 17026 |  | 634.8 | 3865 |  | 686.3 | 30640 |  | 659.2 | 18560 |
| 617.0 | 7103 |  | 650.0 | 3134 |  | 687.9 | 8435 |  | 672.0 | 22953 |
| 637.2 | 21642 |  | 656.0 | 8940 |  | 688.3 | 19857 |  | 686.3 | 26289 |
| 656.0 | 15371 |  | 659.3 | 89873 |  | 708.3 | 6938 |  | 687.9 | 8222 |
| 659.2 | 16057 |  | 666.0 | 8362 |  | 780.3 | 10243 |  | 688.3 | 14783 |
| 2162.9 | 978 |  | 672.0 | 16705 |  | 851.4 | 21268 |  | 780.3 | 8585 |
|  |  |  | 681.3 | 4672 |  | 852.4 | 7045 |  | 827.3 | 6241 |
|  |  |  | 681.9 | 6550 |  | 855.3 | 3557 |  | 851.4 | 21535 |
|  |  |  | 687.9 | 15489 |  | 868.4 | 5672 |  | 852.4 | 6044 |
|  |  |  | 697.3 | 4524 |  | 873.4 | 7274 |  | 868.4 | 3826 |
|  |  |  | 703.9 | 9560 |  | 1105.5 | 4562 |  | 926.4 | 6238 |
|  |  |  | 780.3 | 8903 |  | 1111.5 | 5770 |  | 1105.5 | 4012 |
|  |  |  | 805.4 | 15600 |  | 1121.5 | 7315 |  | 1111.5 | 4672 |
|  |  |  | 807.9 | 2787 |  | 1122.5 | 4525 |  | 1121.5 | 5735 |
|  |  |  | 823.9 | 4391 |  | 1146.5 | 4629 |  | 1122.5 | 2348 |
|  |  |  | 839.8 | 2904 |  | 1162.5 | 5086 |  | 1162.5 | 3655 |
|  |  |  | 851.4 | 10751 |  | 1196.5 | 6655 |  | 1196.5 | 5504 |
|  |  |  | 852.4 | 4015 |  | 1257.5 | 4590 |  | 1257.5 | 3569 |
|  |  |  | 868.4 | 3921 |  | 1385.6 | 13451 |  | 1385.6 | 13292 |
|  |  |  | 877.0 | 6212 |  | 1407.6 | 4175 |  | 1435.5 | 5852 |
|  |  |  | 893.0 | 7981 |  | 1435.6 | 6137 |  | 1459.6 | 6821 |
|  |  |  | 906.4 | 7216 |  | 1459.6 | 7682 |  | 1482.6 | 13900 |
|  |  |  | 908.9 | 4679 |  | 1482.6 | 12856 |  | 1488.7 | 3137 |
|  |  |  | 944.4 | 3736 |  | 1504.6 | 3408 |  | 1548.6 | 2989 |
|  |  |  | 1111.5 | 3349 |  | 1526.7 | 6452 |  | 2162.8 | 4335 |
|  |  |  | 1121.5 | 3820 |  | 2162.8 | 3671 |  | 2184.8 | 5198 |
|  |  |  | 1196.6 | 2651 |  | 2184.8 | 5782 |  | 2192.8 | 3107 |
|  |  |  | 1257.6 | 4537 |  | 2429.0 | 4703 |  | 2428.9 | 5342 |
|  |  |  | 1385.6 | 13764 |  | 2447.0 | 13680 |  | 2431.0 | 2240 |
|  |  |  | 1459.6 | 3643 |  | 2685.0 | 3300 |  | 2447.0 | 17017 |
|  |  |  | 1482.6 | 20285 |  |  |  |  | 2685.0 | 3858 |
|  |  |  | 1500.6 | 5083 |  |  |  |  |  |  |
|  |  |  | 1548.7 | 3502 |  |  |  |  |  |  |
|  |  |  | 1758.6 | 3784 |  |  |  |  |  |  |
|  |  |  | 2162.9 | 9514 |  |  |  |  |  |  |
|  |  |  | 2184.9 | 7683 |  |  |  |  |  |  |
|  |  |  | 2192.8 | 14068 |  |  |  |  |  |  |
|  |  |  | 2200.8 | 4236 |  |  |  |  |  |  |
|  |  |  | 2273.0 | 14495 |  |  |  |  |  |  |
|  |  |  | 2304.9 | 2255 |  |  |  |  |  |  |
|  |  |  | 2447.0 | 8282 |  |  |  |  |  |  |
